# The first purely open access dermatology journal from the British Association of Dermatologists and Wiley

**DOI:** 10.1002/ski2.2

**Published:** 2020-08-07

**Authors:** G. Millington

**Affiliations:** ^1^ Department of Dermatology Norfolk and Norwich University Hospital Norwich UK

I am really excited to be the first Editor in Chief of Skin Health and Disease (SHD), a collaboration between the British Association of Dermatologists (BAD), in our centenary year, and Wiley, the publishers. I thoroughly enjoyed my time as Editor in Chief of Clinical and Experimental Dermatology (CED), from January 2013 to December 2018, and I am now eager for a new challenge. The original BAD journal is the British Journal of Dermatology (BJD), which started in 1888. It was renamed as the ‘British Journal of Dermatology and Syphilis’ in 1917 and reverted to its original name only in 1950. The BJD is one of the oldest and most highly cited international dermatology journals. It publishes the highest‐quality research to advance the understanding and management of skin disease to improve patient outcomes. CED started life as the ‘Transactions of the London Dermatological Society’ in 1911, have previously been known as the ‘Transactions of the St John’s Dermatological Society’. It was transferred to the BAD in 1976 and renamed CED. CED is a highly respected and readable provider of original articles and continuing professional development and CED has been rebranded as the BAD’s education journal.

SHD now joins its older siblings, as the BAD and Wiley’s first fully open‐access (OA) Dermatology journal. Wiley has 20 dermatology titles, by far the biggest grouping of all the major publishers (nearly 50% of the dermatology titles out of all the major publishers). Wiley is an American academic publisher, that was established in 1807. It merged with Blackwell’s (established 1879, Oxford, UK), the original publishers of BJD and CED, in 2007. The BJD and CED have a longstanding relationship with Blackwell’s and more recently Wiley Blackwell, going back to 1978.

This new journal is their first purely OA Dermatology journal. So, what is OA publishing? OA publishing is not new. Many established journals, such as BJD and CED, are hybrid journals, publishing a small proportion of their articles in exchange for an article‐processing charge (APC). This is the hybrid model. For a pure OA journal, the APC will have to be paid for every article, unless the relevant university has an agreement with the publisher to waive the APC. There are agreements currently with many universities in the United Kingdom, Germany, the Netherlands, Austria, Sweden, Norway, Finland, Hungary and currently in the United States, we have agreements with Ohio and Virginia. Authors from these regions will not necessarily have to pay to publish. What are the advantages of publishing OA?

1. Articles are published very quickly
2. Articles are freely accessible to readers
3. Certain article types may be more easily published OA (e.g., negative data)
4. Some funders require it (Wellcome Trust, Gates Foundation)

SHD is a multidisciplinary international open access journal covering all aspects of dermatology including basic science, translational and clinical research. We especially welcome cutting‐edge interdisciplinary and multiprofessional dermatology research. This includes genetics, immunology, pathology, surgery or pure clinical studies, as well as artificial intelligence in dermatology and teledermatology studies. The overarching aim of the journal is to improve patient outcomes by increasing our understanding of skin problems at every stage from disease pathogenesis to its treatment and prevention.

We will publish research articles, reviews, case reports, method papers, protocols, negative studies and pilot studies if the article can demonstrate robust methodology, scientific validity and reliability. All papers will undergo rigorous and fast peer review, as well as rapid publication times.

Welcome to your new journal! For any pre‐submission enquiries contact, shd@bad.org.uk

## CONFLICT OF INTERESTS

No conflict of interests have been declared.

